# Editorial: Natural phytochemicals to enhance animal productivity and health status with low greenhouse gas emissions

**DOI:** 10.3389/fvets.2023.1280611

**Published:** 2023-09-27

**Authors:** Hani M. El-Zaiat, Juan Carlos Ku-Vera, Yosra A. Soltan

**Affiliations:** ^1^Department of Animal and Veterinary Sciences, College of Agricultural and Marine Sciences, Sultan Qaboos University, Muscat, Oman; ^2^Department of Animal and Fish Production, Faculty of Agriculture, University of Alexandria, Alexandria, Egypt; ^3^Facultad de Medicina Veterinaria y Zootecnia, Universidad Autónoma de Yucatán Mérida, Mérida, Mexico

**Keywords:** bioactive components, methane, performance, health, immunity, alternatives

Phytochemical components are widely acknowledged as safe for human consumption, resulting in greater social acceptance compared to chemical feed additives. Phytochemical compounds exhibit more potent mechanisms of action, manipulating the ruminal environment in ways that may circumvent microbial adaptation effects and prevent loss of activity over time. Beyond these benefits, phytochemicals offer antimicrobial, antioxidant, and immune-modulating properties that can enhance animal health, alleviate stress, and even inhibit the production of greenhouse gas emissions (GHG). The concern about GHG emissions from the livestock sector has increased in recent years, as some reports pointed out livestock as one of the significant contributors to these emissions. Key players in methane (CH_4_) production, known as methane-forming archaea or methanogens, maintain crucial syntrophic relationships with other microorganisms within the animal gastrointestinal tract. These archaea utilize secondary metabolites from protozoa and cellulose-fermenting bacteria to prevent hydrogen accumulation in the rumen and maintain proper fermentation processes. Therefore any strategy to reduce ruminal CH_4_ formation needs to consider removing excess metabolic hydrogen from the rumen. The main focus of this topic inclined to invite research ideas to understand the mode of action of phytochemical components and their effects on livestock production, and animal health, considering the practical use for most livestock farmers. This topic comprises 14 articles centered around the utilization of phytochemical components due to their antimicrobial, antioxidant, and immune-modulating attributes. These components were found to have the potential to enhance animal health, mitigate environmental stress, and contribute to the reduction of GHG in animal feeding and nutrition.

## 1. Combining aqueous leaf extracts from plants to reduce GHG originating from ruminant animals

The Research Topic garnered attention with an *in vitro* study that explored various delivery options, including individual and combined forms. For instance, the study investigated the impact of aqueous extracts from *Azadirachta indica* (AZI) and *Cnidoscolus angustidens* (CNA), as well as their 1:1 combination (Mix), on rumen microbial structure and activity. This study, conducted by Elghandour et al., revealed that the combined treatment (Mix) exhibited the most significant CH_4_ reduction, suppressing CH_4_ production by 67.7%, surpassing the effects observed when using AZI or CNA alone. The authors further advocated that the synergistic potential resulting from the combination of both additives contributed to a sustainable decrease in CH_4_ production and other gases (e.g., hydrogen sulfide) while maintaining the production of ruminal short-chain fatty acids (SCFA).

## 2. Supplementation of plant bioactive components to improve colostrum and milk production

Supplementation of *Capsicum oleoresin* (CAP) from 4 weeks prepartum at a dose of 20 mg CAP/kg dry matter to buffalo diets enhanced the lactose and monounsaturated fatty acids in colostrum by 26.5% and 70% respectively. Calf growth performance was positively improved, which suggested that CAP can be a potential prepartum maternal supplement for enhancing newborn buffalo calves' health and performance (An et al.). Feeding dairy cows with red seaweed *Chondrus crispus* supplementation at 6% of the dietary dry matter potentially reduced CH_4_ production by 13.9% with no adverse effects on milk yield and composition (Reyes et al.).

## 3. Essential oils affect the rumen microbial composition and microbial communities

Supplementation of essential oil positively impacted CH_4_ emissions in ruminants. A new essential oil [*Pinus koraiensis* cone (PEO)] was used to reduce CH_4_ emission as one of the potential manipulation strategies (Choi et al.). The administration of PEO at a dose of 1 g/day to growing Korean native goats decreased the ruminal concentrations of total SCFA and ammonia-N, propionate proportion by 14, 2.1 and 46%, respectively, and reduced fungal and prokaryotic microbiota abundance, while did not affect the total bacteria, ciliate protozoa, and methanogen abundance. The oral administration of PEO significantly increased the blood biochemical metabolites, such as albumin, alanine transaminase/serum glutamic pyruvate transaminase, and creatinine by 13%, 15%, and 23%, respectively, while decreasing triglycerides by 45%, thus impacting the fundamental metabolic functions of the liver and kidneys.

## 4. Flavonoids use in beef and dairy cattle diet

The impacts of flavonoids supplementation on an array of factors encompassing performance, antioxidant capacity, rumen fermentation, meat quality, and milk composition in beef and dairy cattle were summarized by Orzuna-Orzuna et al. in a meta a meta-analysis study. Flavonoid supplementations resulted in a pivotal role in enhancing various aspects, such as blood antioxidant capacity, animal performance, nutrient digestibility, and product quality in cattle. The inclusion of flavonoids in animal diets notably led to improvements in meat quality (e.g., reductions in shear force and malondialdehyde content). Flavonoid administration resulted in heightened serum concentrations of superoxide dismutase, glutathione peroxidase, and total antioxidant capacity. This elevation contributed to the facilitation of feed ingestion, nutrient availability, absorption, and utilization, ultimately contributing to improved animal health. For dairy cattle, flavonoids exhibited a dual effect: a reduction in milk somatic cell count and an increase in milk production, protein content, and milk fat content. These favorable outcomes are postulated to be primarily rooted in the stimulation of the digestive tract, physiological-morphological processes, digestive functions, and enzymatic activity. Additionally, flavonoids appeared to induce modifications in the ruminal microbiota, concurrently alleviating the adverse effects of pathogens.

## Author contributions

HE-Z: Conceptualization, Writing—original draft, Writing—review and editing. JK-V: Conceptualization, Writing—review and editing. YS: Conceptualization, Project administration, Writing—review and editing.

